# Repair of the Injured Spinal Cord by Schwann Cell Transplantation

**DOI:** 10.3389/fnins.2022.800513

**Published:** 2022-02-17

**Authors:** Haitao Fu, Die Hu, Jinli Chen, Qizun Wang, Yingze Zhang, Chao Qi, Tengbo Yu

**Affiliations:** ^1^Department of Sports Medicine, The Affiliated Hospital of Qingdao University, Qingdao University, Qingdao, China; ^2^State Key Laboratory Cultivation Base, Shandong Provincial Key Laboratory of Ophthalmology, Qingdao Eye Hospital, Shandong Eye Institute, Shandong First Medical University and Shandong Academy of Medical Sciences, Qingdao, China; ^3^Department of Orthopedics, The Affiliated Hospital of Qingdao University, Qingdao, China; ^4^Key Laboratory of Biomechanics of Hebei Province, Department of Trauma Emergency Center, The Third Hospital of Hebei Medical University, Orthopaedics Research Institution of Hebei Province, Shijiazhuang, China

**Keywords:** spinal cord injury, Schwann cells, neurological disorders, remyelination, nerve regeneration

## Abstract

Spinal cord injury (SCI) can result in sensorimotor impairments or disability. Studies of the cellular response to SCI have increased our understanding of nerve regenerative failure following spinal cord trauma. Biological, engineering and rehabilitation strategies for repairing the injured spinal cord have shown impressive results in SCI models of both rodents and non-human primates. Cell transplantation, in particular, is becoming a highly promising approach due to the cells’ capacity to provide multiple benefits at the molecular, cellular, and circuit levels. While various cell types have been investigated, we focus on the use of Schwann cells (SCs) to promote SCI repair in this review. Transplantation of SCs promotes functional recovery in animal models and is safe for use in humans with subacute SCI. The rationales for the therapeutic use of SCs for SCI include enhancement of axon regeneration, remyelination of newborn or sparing axons, regulation of the inflammatory response, and maintenance of the survival of damaged tissue. However, little is known about the molecular mechanisms by which transplanted SCs exert a reparative effect on SCI. Moreover, SC-based therapeutic strategies face considerable challenges in preclinical studies. These issues must be clarified to make SC transplantation a feasible clinical option. In this review, we summarize the recent advances in SC transplantation for SCI, and highlight proposed mechanisms and challenges of SC-mediated therapy. The sparse information available on SC clinical application in patients with SCI is also discussed.

## Introduction

Spinal cord injury (SCI) is an neurological condition that results in a range of functional impairments including severe motor, sensory, and autonomic dysfunction. Functional recovery after SCI is generally limited. Preclinical research continues to improve our understanding of mechanisms that underlie the pathophysiology of SCI. Currently, various cellular, molecular, and bioengineering strategies have been investigated with the goal of promoting repair and recovery from injury. Cell transplantation offers more advantages than drugs, nerve growth stimulatory factors, or biomaterials for repairing the spinal cord. Cell grafts can provide trophic support, neuroprotection, remyelination, and anti-inflammatory effects, as well as forming permissive tissue bridges for axon regeneration across injured sites, including neural stem cells (NSCs), neural progenitor cells (NPCs), olfactory ensheathing cells (OECs), Schwann cells (SCs), oligodendrocyte progenitor cells (OPCs), mesenchymal stem cells (MSCs), activated macrophages, and induced pluripotent stem cells (iPSCs) (Silva et al., 2014; Assinck et al., 2017a). Compared to other cells used in SCI repair, SCs can be highly purified, and well characterized and are relatively easy to isolate and expand from autologous nerves, which makes them an outstanding cell type for SCI repair (Monje et al., 2021).

SC transplantation for SCI repair has a long history. In the early 1980s, two landmark papers were sequentially published showing that axons from central nervous system (CNS) neurons can grow into peripheral nerve grafts (Richardson et al., 1980; David and Aguayo, 1981), which challenged the traditional concept that neurons in the CNS were incapable of regeneration after injury. In Berry et al. (1988) demonstrated that SCs play a primary role in the regeneration of axons into peripheral nerve grafts when normal or acellular peripheral nerve grafts are transplanted into the CNS. Numerous axons penetrated deeply into the cellular grafts but not the acellular peripheral nerve grafts. Since SCs were known to be pivotal in the regeneration of axons into peripheral nerve grafts, this raised the question of why not transplant purified SCs into the CNS to promote CNS nerve regeneration following injury? In 1981, the first study involving the transplantation of purified SCs into the spinal cord was performed. The study, which was conducted in a focal demyelination mouse model, revealed that SCs could myelinate spinal cord axons (Duncan et al., 1981). More SC-myelinated axons regrew into the cell bridge in a complete transection (Xu et al., 1997) or contusion (Takami et al., 2002) model when SCs were introduced into the injured spinal cord. Recently, substantial progress has been made in the use of SCs for spinal cord repair and improving functional recovery in animal models of SCI (Takami et al., 2002; Pearse et al., 2004b; Biernaskie et al., 2007; Tetzlaff et al., 2011; Deng et al., 2015, 2021; Sparling et al., 2015; Bastidas et al., 2017). However, little is known about the mechanisms by which transplanted SCs promote repair and mediate functional improvements. An understanding of these mechanisms would facilitate development of novel effective interventions to improve functional outcomes after SCI. Therefore, this review focuses on proposed mechanisms of SC transplantation for SCI. In addition, challenges of SC-based therapeutic are also discussed.

## Generation and Characteristics of Schwann Cells

**FIGURE 1:**
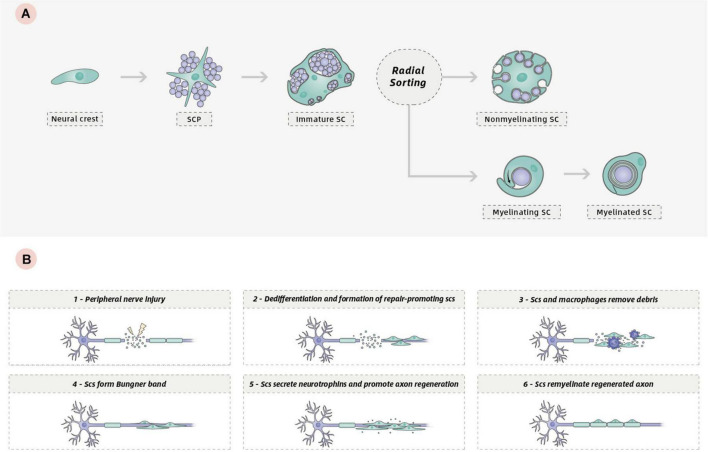
**(A)** Development of the SC lineage: from neural crest cells to myelinating and non-myelinating SCs. **(B)** Schematic of the repair program orchestrated by SCs following peripheral nerve injury.

SCs, including myelinating and non-myelinating SCs in nerve trunks and in nerves generally, are generated from the neural crest, or from crest-like cells (Woodhoo and Sommer, 2008). During early developmental stages, neural crest cells are migratory, proliferative, and multipotent. They generate Schwann cell precursors (S) and many other cell types, including neural and non-neural cells. The appearance of SCPs represents the first step in the generation of SCs from neural crest cells. Similar to neural crest cells, SCPs are also migratory and proliferative and give rise to immature SCs. This represents the second step in the generation of SCs (Woodhoo and Sommer, 2008; Woodhoo et al., 2009; Monk et al., 2015). Distinguished from SCPs, immature SCs cease migrating, remain proliferative, and form an immature basal lamina (Jessen and Mirsky, 2005). At birth, the immature SCs differentiate into either myelinating or non-myelinating SCs through radial sorting, a process by which these immature cells sort individual axons depending on the type of axon they engage with. Myelinating SCs ensheath one axon segment and subsequently spiral their membrane many times to form a compact myelin sheath. By contrast, non-myelining SCs, also referred to as Remak SCs, ensheath multiple small caliber axons without making myelin (Figure 1A). A detailed description of SC development and the molecular mechanisms regulating SC generation and myelination in the peripheral nervous system (PNS) has been provided elsewhere (Woodhoo and Sommer, 2008; Pereira et al., 2012; Monk et al., 2015; Muppirala et al., 2020).

One key characteristic of the biology of SCs is their remarkable plasticity, which confers a high regenerative capacity for the PNS. Following PNS injury, progressive breakdown and clearance of distal axons termed Wallerian degeneration occurs to create a permissive environment for regeneration. During the process, mature SCs can dedifferentiate to a phenotype related to that of the immature SCs, which promotes axonal regeneration (Jessen and Mirsky, 2008). This phenotypic conversion is associated with the downregulation of several promyelinating genes and the upregulation of growth-promoting genes (Chen et al., 2007; Balakrishnan et al., 2016). In injured adult nerves, repair-promoting SCs and invading macrophages degrade and remove damaged axons and myelin debris (Gomez-Sanchez et al., 2015). Then, SCs extend long parallel processes and align in tracts called Bungner band to guide axon regrowth (Arthur-Farraj et al., 2012; Jessen and Arthur-Farraj, 2019; Nocera and Jacob, 2020). Finally, repair SCs redifferentiate into myelinating SCs to remyelinate the regenerated axon (Figure 1B). Another key characteristic of SCs is their ability to produce extracellular matrix (ECM) components and a variety of neurotrophins that can support survival of damaged neurons and promote axon regeneration, including nerve growth factor (NGF), brain-derived neurotrophic factor (BDNF), ciliary neurotrophic factor (CNTF), and neurotrophin-3 (NT-3), as well as the expression on their surfaces of various cell adhesion molecules (Bunge, 1994; Wilson et al., 2020). All these characteristics allow SCs to promote repair in PNS or CNS injury.

## Function and Mechanism of Schwann Cells in Spinal Cord Injury Repair

### Axon Regeneration

**FIGURE 2:**
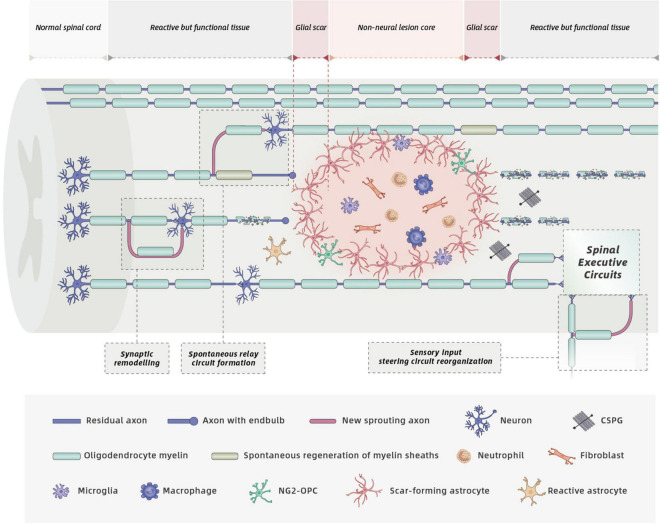
Cell biology and spontaneous recovery after SCI. Mature SCI lesions have three different compartments: (1) a central non-neural lesion core, consisting of stromal cells, meningeal fibroblasts, and pericytes. (2) a narrow glial scar, consisting of reactive astrocyte and microglia, that intimately surround the lesion core, and (3) a large surrounding zone of functional neural tissue (O’Shea et al., 2017; Courtine and Sofroniew, 2019). SCI triggers complex pathological events including considerable loss of axons, neurons, and oligodendrocytes, infiltration of circulating immune cells, release of detrimental molecules by multiple cells, and glial scar formation. These processes within distinct SCI lesion compartments contribute to functional impairments. Spontaneous recovery can occur due to spontaneous circuit reorganization, spontaneous regeneration of myelin sheaths (produced by both oligodendrocytes and endogenous SCs) and spinal cord automaticity. However, spontaneous axonal regeneration is hampered by the increased deposition of CSPGs secreted by activated cells, and increased production of myelin-associated inhibitory molecules by the gradual degradation of injured oligodendrocyte myelin.

A key pathological event of SCI is axonal severance induced by the initial trauma (Blesch and Tuszynski, 2009; Lu et al., 2014). Even though spontaneous relay circuit formation takes place in spared neural tissue for anatomically incomplete SCI (Bareyre et al., 2004; Blesch and Tuszynski, 2009; Rosenzweig et al., 2010; Takeoka et al., 2014; O’Shea et al., 2017; Hutson and Di Giovanni, 2019; Figure 2), the degree of functional recovery induced by this spontaneous process is limited. Thus, a logical repair strategy would be to first promote regeneration of severed axons across large non-neural lesion cores to restore neural connectivity.

#### Trophic Factors

Injured axons fail to regrow through the non-neural lesion core where pericytes and fibroblast lineage cells produce various axon-inhibitory molecules. However, it seems unreasonable to attribute the failure of axon regeneration solely to the presence of these inhibitors. Transplanted fibroblasts promote substantive axon regeneration into lesion sites only when they are genetically modified to produce axon-stimulatory growth factors (Blesch and Tuszynski, 2007). Delivery of growth factors required for sensory axon growth during development supports regrowth of sensory axon into lesion cores (Anderson et al., 2016). Therefore, continued delivery of growth factors would be a promising strategy for sustaining axon regrowth following SCI.

**FIGURE 3:**
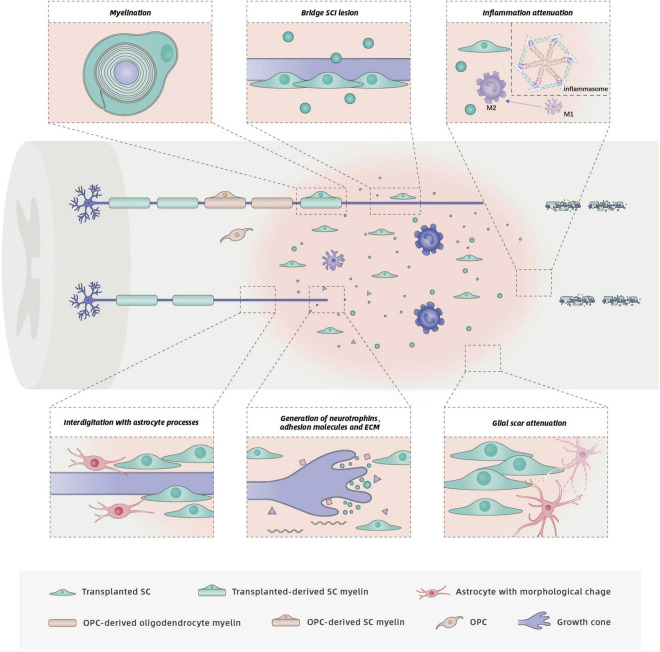
Functions and mechanisms of SCs in SCI repair. The injured spinal cord schematic illustrates potential benefits of SC transplantation, including support of axon growth, remyelination, glial scar attenuation, and related inflammation attenuation. Neurotrophins secreted by SCs bind to specific receptors on axons, which promotes axon regrowth. Although astrocytes prevent SC migration, survival and integration into the normal white matter, the interdigitation of SCs with astrocyte processes has also been observed. The interdigitation of transplanted SC processes with astrocyte processes at the rostral host spinal cord/SC bridge interface results in entry of regenerated axons to the SC bridge. Transplanted SCs form new myelin sheaths around regenerated or spared demyelinated axons. Endogenous OPCs in the spinal cord may differentiate into new oligodendrocytes and PNS-like SCs, both of which produce myelin sheaths. Transplanted SCs also exert neuroprotection by modulating related inflammation.

The transplanted SCs within a lesion core secrete a variety of axonal growth-promoting factors (Figure 3), including NGF, BDNF, CNTF, and NT-3 (Bunge, 1994; Alsmadi et al., 2018). By binding to specific receptors, these factors activate the downstream signaling cascades involved in the axonal outgrowth of both sensory and motor fibers in the CNS (Keefe et al., 2017). Different populations of neural axons have different sensitivities to different neurotrophins. NGF mainly stimulates regeneration of sensory axons, whereas BDNF primarily promotes the sprouting and regeneration of motor axons. NT-3 has a positive effect on both sensory and motor axonal growth after SCI (Keefe et al., 2017). Compared with normal SCs, more axons were observed across the transected spinal cords of adult rats that received SCs genetically modified to secrete increased amounts of BDNF (Menei et al., 1998) or GDNF (Deng et al., 2013). Although functional outcomes were not assessed, a combination of BDNF and NT-3 exerted synergistic effects, and significantly more myelinated fibers regenerated into SC grafts in a rat model of acute transection (Xu et al., 1995).

#### Adhesion Molecules

In addition to secreting neurotrophins, SCs express adhesion molecules on their surfaces (e.g., L1 and NCAM) and produce ECM components (e.g., laminin, fibronectin, and collagen) (Bunge, 1994, 2016; Mirsky et al., 2002; Figure 3). These unique characteristics of SCs also support axonal regeneration. Neural cell adhesion molecule (NCAM) and the L1 family of adhesion molecules have important roles in axonal outgrowth, myelination, and synapse formation during nervous system development and regeneration (Maness and Schachner, 2007; Dityatev et al., 2008; Kataria et al., 2016). Deletion of NCAM in adult mice reduced axonal regrowth/sprouting at the site of injury and impaired function recovery after SCI (Saini et al., 2016). L1 promotes neurite growth by neutralizing inhibitors of axonal growth in the CNS. Application of small-molecule L1 agonists stimulated neurite outgrowth and functional recovery in mouse models of femoral nerve injury or SCI (Kataria et al., 2016). Enhanced recovery after SCI in the adult rodent nervous system was also observed following application of soluble cell adhesion molecule L1-Fc (Roonprapunt et al., 2003) or recombinant L1 Fab fragment directed against L1 (Loers et al., 2014) and L1-overexpressing SCs or stem cells (Chen et al., 2005; Lavdas et al., 2010; Cui et al., 2011).

#### Extracellular Matrix

Nerve regeneration following injury in the PNS is more successful compared to that in the CNS; this is due, in part, to the generation of ECM components that provide structural strength as well as substrates for the adhesion and outgrowth of regenerating axons. Laminin and fibronectin are upregulated following peripheral nerve injury (Gardiner, 2011). Therefore, considerable effort is focused on using ECM proteins to provide a permissive environment for axonal regeneration following SCI. King et al. (2010) demonstrated that injectable forms of fibronectin and/or fibrin supported axonal growth 4 weeks after injection into an experimental knife-cut cavity in the rat spinal cord. Takeda et al. (2015) showed that laminin guided axon growth through scar tissue in a goldfish model of SCI. Laminin was typically restricted to blood vessels in the spinal cord and to the endogenous SCs present in the spared rim of the lesion core after SCI (Assinck et al., 2020). By contrast, laminin was extensively expressed in the spinal cord of SC-treated animals (Assinck et al., 2020).

#### Scar Attenuation

Another proposed mechanism for the enhanced axon growth following SC transplantation is likely to be modification of glial scars which is generally thought to be a major block for axonal regeneration (Figure 3). Skin precursor-derived SCs were shown to mitigate glial scar formation, displayed predominant rostro-caudal orientation, formed cellular conduits to bridge the SCI lesion, and improved locomotor outcomes (Assinck et al., 2020). Although the complex roles of the glial scar in CNS axon growth are unclear, sensory or motor axons must penetrate the caudal or rostral “wall” of glial scars to access the SC bridges onto which axons can grow. More recent interventions have therefore focused on the SC implant/host spinal cord interface with the goal of fostering axon regeneration across the cysts and scars. Following SC transplantation, an irregular interface was formed due to the interdigitation of astrocyte processes with implanted SC processes, which resulted in more regeneration of axons into the SC bridges rather than a distinct border (Williams et al., 2015). Furthermore, the number of axons in cellular bridges was in direct proportion to the number of astrocyte processes that extended into the bridges (Williams et al., 2015). Thus, the morphological change in astrocytes induced by the interplay between astrocytes and SCs likely responsible for the regrowth of axons into the lesion.

### Myelin Repair

A spared rim of axons remains at the periphery of the spinal cord lesion in both rodent (Basso et al., 1996) and human SCI (Kakulas, 2004). The remaining axons lose their myelin sheaths as a result of oligodendrocyte death induced by secondary injury. Loss of oligodendrocytes and demyelination is believed to further impair the conductive capacity and neural functional recovery after SCI (McDonald and Belegu, 2006). Spontaneous regeneration of myelin sheaths, termed remyelination, is achieved by endogenous oligodendrocytes and SCs in animal and human SCI (Zawadzka et al., 2010; Plemel et al., 2014; Assinck et al., 2017b; Figure 2). OPCs migrate to the lesion area and differentiate into new oligodendrocytes that generate new myelin sheaths. Interestingly, demyelinated axons can also be remyelinated by SCs, although the origin of these CNS-resident SCs is still debated (Blight and Young, 1989; Zawadzka et al., 2010; Assinck et al., 2017b; Chen et al., 2021). However, the persistent dysfunctional neural conductivity suggests that spontaneous remyelination by endogenous oligodendrocytes and SCs after SCI seems insufficient (Nashmi and Fehlings, 2001). Therefore, the transplantation of SCs aims to replace lost oligodendrocytes incurred by SCI, to boost the remyelination of remaining axons in spared white matter and to promote the recovery of electrical impulse conduction.

#### Schwann Cell Remyelination in Central Nervous System

Transplanted SCs tightly associate with, align along, and myelinate host axons when introduced into the injured rat spinal cord (Figure 3). More axons surrounded by typical peripheral type (SC) myelin were identified in SC-treated animals compared to medium-injected animals (Takami et al., 2002; Biernaskie et al., 2007; Sparling et al., 2015; Assinck et al., 2020). Approximately 73% of the transplanted SCs were myelinating in the chronically injured spinal cord (Assinck et al., 2020). In a contusion SCI model, robust SC myelination was reported at 12 weeks after transplantation (Wang and Xu, 2014). Endogenous SCs were also observed myelinating host axons following SCI (Biernaskie et al., 2007; Sparling et al., 2015). Myelination by endogenous, centrally derived SCs can be detected 3 weeks after lesion induction (Zawadzka et al., 2010). Quantitative analysis of SC-myelinated axons in both the grafts and spared tissue rim revealed that 53% of axons were myelinated by endogenous SCs after contusive thoracic SCI (Biernaskie et al., 2007). Approximately 40% of SC myelin was generated from endogenous SCs in the partially injured cervical spinal cords of SC-treated rats, whereas little SC myelin was observed in a medium-injected group (Sparling et al., 2015). Therefore, in addition to myelinating axons, transplanted SCs recruit endogenous SCs to myelinate the demyelinated axons (Assinck et al., 2020).

#### Oligodendrocyte Progenitor Cell-to-Schwann Cell Differentiation

Why are peripheral SCs present in the CNS? Researchers once thought that SCs entered the CNS from PNS sources, but this paradigm has been challenged by several recent studies. Using a chemically induced demyelinated model, Zawadzka et al. (2010) showed that OPCs residing in the adult CNS produced most of the SCs during CNS remyelination. Only a small decline in the number of SCs was observed in the injured spinal cord after removal of the bilateral dorsal roots, suggesting that the majority of SCs are generated from a central source (Bartus et al., 2016).

The molecular mechanism underlying the trans-differentiation of OPCs into PNS-like SCs following SCI remains unclear, however, it may involve bone morphogenetic protein (BMP) (Talbott et al., 2006). *In vitro*-cultured reactive astrocytes from the contused spinal cord expressed high levels of BMP, which inhibited differentiation of OPCs into oligodendrocytes (Wang et al., 2011b). Consistent with this finding, inhibition of BMP signaling is sufficient to block OPC-to-SC differentiation in lesions (Talbott et al., 2006). Another factor proposed to drive the OPC-to-SC transition may be the growth factor neuregulin-1 (Nrg1) (Bartus et al., 2016, 2019). SCs were absent from the contused spinal cord in adult mice with conditional ablation of Nrg1. However, these studies did not directly demonstrate that Nrg1 drives the OPC-to-SC transition, and only showed that Nrg1 is necessary for the transition, likely because SCs require Nrg1 for survival (Dong et al., 1995). The presence of myelinating SCs in the CNS suggests a reduction in the number of myelinating oligodendrocytes from OPCs. Whether SC myelin in the CNS and oligodendrocyte myelin have equally important functions is not known. Neither a compensatory increase in oligodendrocyte remyelination nor a better functional recovery was observed in the context of SC absence (Bartus et al., 2016, 2019). Thus, promoting OPC-to-SC differentiation is likely to be a promising therapeutic strategy (Duncan et al., 2020).

#### Motor Exit Point Glia

In addition to oligodendrocytes and SCs in nervous systems, MEP glia is a recently discovered type of myelinating cells that myelinate spinal motor root axons (Kucenas et al., 2009; Smith et al., 2014). MEP glia originate from the CNS, share a common progenitor with oligodendrocytes, reside outside of the spinal cord and function in the PNS (Fontenas and Kucenas, 2018). Numerous studies showed that MEP glia could regulate glial migration across transition zones, establish and maintain CNS/PNS boundary (Smith et al., 2014; Morris et al., 2017). Although their identity (a central or a peripheral or a hybrid glial cell) is not uncovered, they do myelinate axons, which raises a question that whether these unidentified, centrally derived glial cells could myelinate the demyelinated axons when transplanted to spinal cord after SCI. With single-cell RNA sequencing development, the characteristics of this unique glial population will be elucidated, and may be relevant also in spinal regeneration like SCs.

### Neuroprotection

Neuroprotection is a treatment option for many CNS disorders, including neurodegenerative diseases, stroke, and traumatic brain/spinal cord injury. In the context of SCI, neuroprotection is believed to be one of the mechanisms by which transplanted SCs exert a beneficial effect on the injured cord, because transplanted SCs reduced cavitation and increased sparing around the lesion compared with controls (Takami et al., 2002; Pearse et al., 2004a; Biernaskie et al., 2007; Schaal et al., 2007; Assinck et al., 2017a,^2020^; Bastidas et al., 2017). However, attributing the reduction of cavitation or increase of sparing tissue to neuroprotection is not plausible, given that the axon regeneration and myelination by transplanted SCs might increase the amount of spared tissue around the injury site (Assinck et al., 2017a).

Neuroprotection is defined as the relative preservation of neuronal structure and/or function (Casson et al., 2012). The bona fide neuroprotective effect of SCs is most likely a result of their secretion of growth factors, which support the survival of injured host cells. A twofold increase in the numbers of preserved NeuN + neurons around the lesion was observed in an SC-transplanted group (Schaal et al., 2007). In addition, transplanted SCs have been shown to exert neuroprotection by modulating inflammation (Pearse et al., 2018; Mousavi et al., 2019). Similar to immunomodulatory drugs that selectively inhibit or enhance components of the innate immune response (Schaal et al., 2012; Kobayashi et al., 2013; Fehlings et al., 2017; Gensel et al., 2017) and other exogenous cells that alter the polarization or activity of immune cells (Mosher et al., 2012; Zanier et al., 2014; DePaul et al., 2015), SC delivery also provides immunomodulatory effects to indirectly improve neuron survival after SCI. In a rat model of thoracic contusive SCI, SCs were introduced into the injured spinal cord at 1 week following injury. The introduction of SCs significantly reduced the numbers of CD11b+, CD68+, and Iba1 + cells and enhanced the transition of immune cells from a pro-inflammatory to anti-inflammatory phenotype (Pearse et al., 2018; Figure 3). An important component of the innate immune system is the inflammasome, which is known to be involved in the pathogenesis of several CNS diseases (Tschopp and Schroder, 2010). Activation of the inflammasome in CNS injury is responsible for inflammation propagation and neuronal cell death. Administration of SCs attenuated the activity of inflammasome complexes and related inflammatory circuits while improving motor function (Mousavi et al., 2019).

## Schwann Cell Transplantation for Clinical Application

Extensive preclinical data indicate the efficacy of SC transplantation for rodent SCI repair. However, any decision on the clinical application of SCs must be made with caution and prudence. Safety is the first consideration for the clinical use of SCs.

The establishment of techniques for human SC isolation and purification has enabled the generation of large numbers of SCs from patients’ sural nerves within a limited therapeutic time window (Casella et al., 1996). Use of human autologous SCs from the adult peripheral nerves circumvents the immune response and ethical problems and minimizes the risk of disease transfer. More importantly, human SCs have been shown to be safe and do not induce glioma formation when transplanted into the injured spinal cord of adult rodents (Guest et al., 2013; Bastidas et al., 2017; Monje et al., 2021). Although tumor formation by transplanted rodent SCs has been reported for adult skin-derived and postnatal nerve derived SCs (Langford et al., 1988; May et al., 2018), no evidence of tumor formation of the human SC transplants in spinal cord lesions was found in preclinical investigations of donor nerve-derived human SCs (Bastidas et al., 2017). Several clinical trials (Table 1) have also confirmed their safety and feasibility for the treatment of SCI, although little functional improvement was observed (Saberi et al., 2008, 2011; Zhou et al., 2012; Anderson et al., 2017; Gant et al., 2021). Saberi et al. (2011) administered SCs into 33 patients with chronic SCI (24 with thoracic injuries and 9 with cervical injuries). Autologous SCs purified from the sural nerves were injected into the cavity, and the patients were followed up for 2 years. Some patients showed improvements in motor, sensory, and sphincter functions. No adverse effects were observed 2 years after SC treatment. Zhou et al. (2012) also reported that autologous SC transplantation for the treatment of SCI resulted in signs of improvement in autonomic and sensorimotor function. In addition to SC transplantation alone, a combination of SCs with other cell types also indicated no undesirable effects in human participants (Chen et al., 2014; Oraee-Yazdani et al., 2016).

**TABLE 1:** Clinical trials of Schwann cell transplantation (alone or together with other cell types) for spinal cord injury.

Study		Country	Age, number of patients	Injured level and severity	Transplanting time after injury	Cell dose	Cell delivery	Follow-up period	Outcomes
Saberi et al. (2008)		Iran	22–43, 4	T6-T9 ASIS A or C	28–80 months	3–4.5 × 10^6^ cells in 300 μl	5–6 locations on each side rostral or caudal to the lesion	1 year	No adverse effects, no improvement of sensorimotor, sphincter and sexual function
Saberi et al. (2011)		Iran	23–50, 33	Thoracic or cervical ASIA A or B	Mean 4.1 years	3 × 10^6^ cells in 300 μl	3 different locations within cavity	2 years	No neurological worsening, no increase in syrinx size, no tumor formation, significant improvement of light touch sensory, improvement of sphincter abilities in some patients, no significant increase in FIM and FAM scores
Zhou et al. (2012)		China	7–44, 6	C5-T12 ASIA A-C	1 week to20 months	4–6 × 10^6^ cells in 200 μl	6–7 locations on each side of spinal cord	5–8 years	Increase in ASIA and FIM scores, improvement of automatic function, increase in latency period and wave amplitude of SSEPs and MEPs
Chen et al. (2014)	Alone	China	22, 1	C4-C6, ASIA A	7 years	10^6^ cells in 50 μl	In the dorsal midline of the spinal cord, above and below the lesion	6 months	Functional neurological improvements, improvements on the electrophysiological test
	With OECs		39, 1	C5-C7 ASIA A	5 years	5 × 10^5^ SCs and 5 × 10^5^OECs in 50 μl			
Oraee-Yazdani et al. (2016)	With BMSCs	Iran	22–45, 6	C7-T11 ASIA A	28–62 months	10^6^ SCs and 10^6^ BMSCs in 2 ml	Though cerebral spinal fluid	30.6 ± 4.7 months	No neoplastic tissue overgrowth, Increase in ASIA scores and indexes of UDS in one patient, No motor score improvement
Anderson et al. (2017)		United States	24–41, 6	T1-T6 ASIA A	4–7 weeks	5 × 10^6^ cells in 50 μl or 10 × 10^6^ cells in 100 μl or 15 × 10^6^ cells in 150 μl	Into injury epicenter	1 year	No surgical, medical, or neurological complications, no adverse events, no additional tissue damage, mass lesion, or syrinx formation, no clear efficacy on functional recovery
Gant et al. (2021)		United States	18–65, 8	C5,C6,T2,T10, T11 ASIA A-C	1–15 years	5 × 10^6^ cells in 500 μl or until cavity was filled with cell suspension	Into cystic cavity	2 years	No serious adverse events related to sural nerve harvest or SC transplantation; reduction in cyst volume; improvement in motor and sensory function; improvement in neurological level of injury

*ASIA, American Spinal Injury Association; FIM, Functional Independence Measure; FAM, Functional Assessment Measure; SSEPs, somatosensory evoked potentials; MEPs, motor evoked potentials; OECs, olfactory ensheathing cells; BMSCs, bone marrow mesenchymal stem cell; DUS, urodynamic study.*

A phase I clinical trial approved by the Food and Drug Administration (FDA) (NCT01739023) evaluated the safety of human autologous SC transplantation in six adult participants with subacute thoracic SCI. Autologous SCs were harvested from a sural nerve of each patient within 30 days after SCI, cultured *in vitro*, and then introduced into the lesion epicenter within 72 days after SCI. Safety was assessed at 12 months post-transplantation. No surgical, medical, or neurological complications and no additional spinal cord damage, mass lesion, or syrinx formation were observed (Anderson et al., 2017). Another phase I clinical trial (NCT02354625) with the goal of evaluating the safety of human autologous SC transplantation in chronic SCI (4 thoracic and 6 cervical SCIs) conducted by the same team at the Miami Project to Cure Paralysis were also completed. No serious adverse events related to sural nerve harvest or SC transplantation were reported and magnetic resonance imaging (MRI) revealed a reduction in cyst volume (Gant et al., 2021). Importantly, one patient experienced a 4-point improvement in motor function, a 6-point improvement in sensory function and a 1-level improvement in neurological level of injury (Gant et al., 2021). Relatively few patients were enrolled in these clinical trials (a total of 57 patients), although all reported no adverse events related to SC-therapy. More clinical trials are needed to confirm the safety and efficacy of human autologous SC transplantation for SCI.

The source of therapeutic human SCs is another consideration for the clinical use. In addition to autologous human peripheral nerve, stem cell is another potential source of human SCs, especially induced pluripotent stem cells (iPSCs). Many limitations that surround the use of embryonic stem cells or other stem cells are overcome by iPSCs. Several studies demonstrated that iPSCs could differentiate into SCs via an intermediate neural crest stem cell stage (Wang et al., 2011a; Okawa et al., 2013). However, no SCI clinical trials have started using iPSC-derived SCs, largely because of potential safety concerns, the risks of tumor formation or infected (Huang et al., 2020). To mitigate the complications, iPSC-derived SCs must be extensively genetically screened and any undifferentiated cells need to be detected by robust methods to improve purity of SCs in the final cell population for clinical cell therapy.

## Challenges

### Low Survival Rate

SC transplantation without additional interventions has a limited therapeutic effect on SCI. Transplanting SCs alone does not improve the Basso, Beattie, and Bresnahan (BBB) scores in rats, presumably because of the low survival rate post-implantation as well as the limited ability of the cells to migrate into host tissue. Over 80% of transplanted SCs disappeared within the first week after transplantation as a result of hypoxia, inflammation, and the immune response within the injury site (Hill et al., 2007; Pearse et al., 2007). In addition, cell membrane damage during injection, cell leakage out of the lesion, and lack of ECM within the lesion also contribute to significant transplanted cell loss and death (Hill et al., 2007; Amer et al., 2017). Less than 5% of injected SCs survive 1 month after transplantation (Marquardt et al., 2020), which may result in limited functional outcomes. Unfortunately, human SCs have a more poor survival rate in contusion injuries with a low proliferation rate when compared to rodent SCs (Bastidas et al., 2017). Many methods are employed to improve the long-term survival of transplanted SCs within the injured spinal cord (Guo et al., 2007; Moradi et al., 2012; Enomoto et al., 2013); however, their effects are unsatisfactory. Therefore, a primary goal is to promote the survival of transplanted SCs in the injured spinal cord to maximize their SCI-repair efficacy. Small molecule compounds are screened to enhance SC survival through reducing oxidative stress, including 17β-estradiol and calpain inhibitor MDL28170 (Hill et al., 2010; Siriphorn et al., 2010). Activating adaptive transcriptional pathways by retroviral expression of hypoxia inducible factor 1 alpha (HIF-1α) in SCs increased SC survival by 34.3% (David et al., 2020). Recently, different biomaterials have also been developed to support survival of transplanted SCs (Lee et al., 2017; Liu et al., 2017a; Wu et al., 2018). A designer, injectable hydrogel resulted in a sevenfold increase in the survival of transplanted SCs with functional recovery compared to the control group during SCI treatment (Marquardt et al., 2020). In a rat model of thoracic contusion, Cerqueira et al. (2018) implanted SCs in acellular injectable peripheral nerve matrix (iPN) into an SCI lesion. The iPN matrix supported SC survival and contained twice as many SC-myelinated axons as Matrigel grafts (Cerqueira et al., 2018).

### Poor Migration

The limited effect of SC transplantation alone is due, in part, to the poor migration of transplanted SCs into host tissue (Hill et al., 2007; Pearse et al., 2007). The molecular mechanisms underlying this poor migration of transplanted SCs is not well understood. Myelin associated glycoprotein (MAG), one of the axonal growth-inhibiting molecules, was shown to inhibit SC migration and induce their death via γ-secretase-dependent cleavage of neurotrophin receptor p75 (Chaudhry et al., 2017). The poor migration of transplanted SCs into the adult CNS is believed to lead to the formation of the SC implant/host tissue interfaces. Although many regenerated descending axons grow into the SC implants from the rostral interface, very few exit the caudal interface and subsequently re-enter the distal host spinal cord. Comparison of the two interfaces showed more chronic and inhibitory conditions at the caudal interface (Wiliams and Bunge, 2012). The extremely slow growth rate of regenerated axons (∼1 mm/day) (Steward et al., 2003) delays their reaching the caudal interface. During this process, increased host astrocyte response and CSPG deposition occurs at the caudal interface (Plant et al., 2001; Lakatos et al., 2003), resulting in generation of a relatively non-permissive environment compared to that at the rostral interface. CSPG digestion with chondroitinase ABC secreted from transduced SCs aided glial integration, enabled more axons to exit from the SC-astrocyte boundary (Warren et al., 2020). Many other efforts were made to enhance the migration of SCs in the astrocytic environment by modifying the characteristics of SCs by altering microRNA levels, or by stimulating the production of polysialic acid (PSA) induction. Overexpression of microRNA-124 in SCs downregulated Krox 20 expression in transplanted cells, inhibited the expression levels of GFAP and p-STAT3 protein in host astrocytes, and ultimately promoted integration of SCs with astrocytes (Li et al., 2020). PSA-modified SCs migrated across the lesion for distances up to 4.4 mm within adjacent host tissue. This was accompanied by significant growth of axons caudal to the lesion (Ghosh et al., 2012). Magnetizing SCs with superparamagnetic iron oxide nanoparticles under a magnetic field effectively enhanced migration of SCs into astrocytic regions (Xia et al., 2016). Besides strategies for improving migratory capacity of SCs, improving the axonal outgrowth rate to enable axons to quickly cross the caudal interface and re-enter the distal spinal cord before generation of non-permissive conditions at the caudal interface may also promote recovery from SCI.

### Limited Regeneration of Corticospinal Axons

Most studies have reported little corticospinal axon regeneration into the SC implant (David and Aguayo, 1981; Richardson et al., 1984; Martin et al., 1996), possibly due to the lower intrinsic growth capacity of corticospinal neurons (CSNs) (Tuszynski and Steward, 2012). Promoting robust corticospinal axon regeneration is essential given the importance of corticospinal function in humans (Deumens et al., 2005; Lemon, 2008; Wang et al., 2017). The administration of SC-derived neurotrophins or other growth factors, such as BDNF, CNTF, and insulin-like growth factor 1 (IGF1), promotes the growth of axons of cultured neonatal CSNs rather than mature CSNs in adults, which suggests that mature CSNs have limited responsiveness to growth factors compared to immature CSNs (Lu et al., 2001; Ozdinler and Macklis, 2006; Hollis et al., 2009). Osteopontin (OPN) sensitizes the response of adult corticospinal neurons to these growth factors. Combining any of the growth factors with OPN promotes robust corticospinal axon regeneration (Duan et al., 2015; Bei et al., 2016; Liu et al., 2017b). Therefore, combinatorial treatment of SC transplantation and OPN in SCI models would likely be more effective promoting corticospinal tract -dependent functional restoration in adults. In addition to OPN, SC transplantation in combination with other treatments also improved SC survival and migration within the injured spinal cord, and increased the axonal growth capacity, which enhanced axon (including corticospinal axon) regeneration into SC bridges beyond the caudal interface; this was accompanied by functional improvement (Pearse et al., 2004b; Fouad et al., 2009; Bunge and Wood, 2012; Ghosh et al., 2012; Wiliams and Bunge, 2012; Bunge, 2016). Given the disadvantages of SC transplantation alone, as well as the multifaceted pathophysiologic changes of SCI, combinatorial therapeutic strategies for SCI repair are necessary. Such multiple-target combinatorial strategies are showing promise for treatment of SCI (Kanno et al., 2014; Bunge, 2016).

## Conclusion

SCs have potential for SCI treatment because of their capacity to promote axon regrowth and myelination within the injured spinal cord. Numerous studies on SC therapy for SCI have reported encouraging results in animal models, and some SC therapies have been the subjects of phase I clinical trials. However, none has been approved for clinical use in SCI patients, possibly due to the complex pathophysiologic mechanisms of SCI and the marked differences between the animal and human spinal cord. Substantial questions and challenges remain in the field of spinal cord regeneration. A better understanding of SCI neuropathology and reparative mechanisms will promote translation of SC therapies from basic research to clinical practice. Combination therapeutic strategies improve the efficacy of SC transplantation for SCI. In the future, patients with SCI will likely be treated using combinations of SC transplantation and other repair-supporting strategies to maximize functional recovery. Moreover, novel methods of treating SCI are needed to improve the quality of life of patients. Although achieving complete recovery after SCI is a challenge, SC therapy shows promise. It is important to remember that even a small improvement in function may have a large impact on a patient’s life.

## Author Contributions

HF and TY: conception and design of the study. HF, DH, CQ, JC, and TY: drafting and revision of manuscript. HF, DH, CQ, QW, and JC: design and drawing of figures. YZ, CQ, and TY: supervision. All authors contributed to the article and approved the submitted version.

## Conflict of Interest

The authors declare that the research was conducted in the absence of any commercial or financial relationships that could be construed as a potential conflict of interest.

## Publisher’s Note

All claims expressed in this article are solely those of the authors and do not necessarily represent those of their affiliated organizations, or those of the publisher, the editors and the reviewers. Any product that may be evaluated in this article, or claim that may be made by its manufacturer, is not guaranteed or endorsed by the publisher.
